# Nanoscale Advances: three years on

**DOI:** 10.1039/d1na90115j

**Published:** 2021-12-08

## Abstract

In this Editorial, we highlight our achievements of the last three years, our core values, and tell you about our aims for the future.
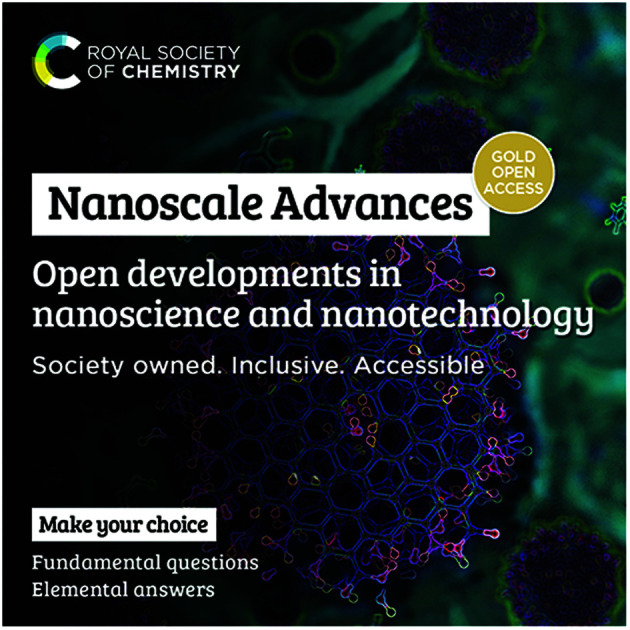

Welcome to issue 1 of our fourth volume of *Nanoscale Advances*. We have been thrilled to see your support of the journal ever since it was launched in 2018 and the first issues published in 2019. We wanted to take a moment to thank you, our readers, authors, and reviewers, for helping *Nanoscale Advances* become a valued home for good quality, reproducible new work, accessible to a global, interdisciplinary network of nanoscale researchers.

## Journal by numbers

**Fig. 1:**
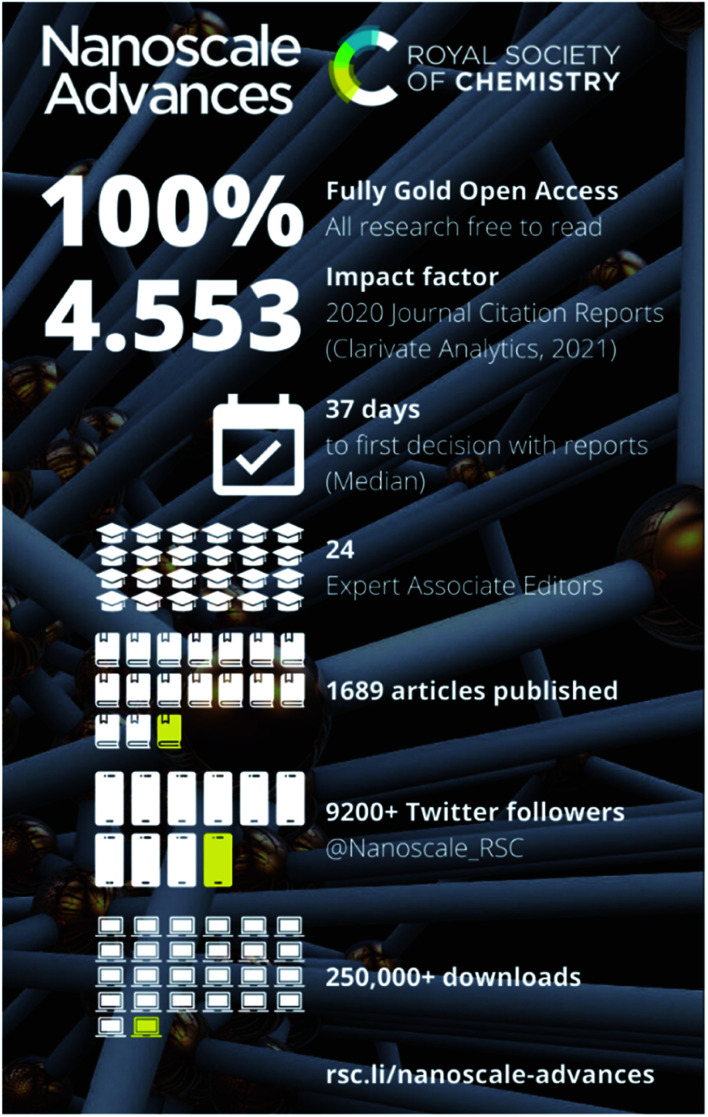
A breakdown of metrics for *Nanoscale Advances* since it was launched.

Fig. 1 shows some of the most recent metrics for the journal.^1^ The Royal Society of Chemistry is a signatory of the San Francisco Declaration on Research Assessment (DORA)^2^ and we recognise that the impact factor is something of a crude measure. However, it remains an important metric to many in our community, thus we are excited to let you know that in June 2021 *Nanoscale Advances* received its very first partial impact factor, embedding our position within the nanoscience publishing community.^1^

The journal has grown steadily over the last three years, publishing 501 articles in 2019, 564 articles in 2020 and we expect to publish more than 600 in 2021. Our authors come from 75 different countries across the globe, and we aim to provide a consistently fair and efficient publishing service to everyone. The median time from submission to first decision over the last 3 years is 37 days, so you can expect a fast but rigorous experience when you submit to *Nanoscale Advances*.

## Gold open access and article processing charges

We recognise that many funding bodies and institutions now require authors to publish in an open access journal. The demand for open access has grown considerably over recent years, and the Royal Society of Chemistry aims to shape the future of open access publishing and maximise the visibility of research in the chemical sciences and related areas.

As a society publisher, our mission is to disseminate chemical knowledge to the largest audience possible, to the benefit of the global scientific community and we aim to do this by focusing on delivering high-quality impactful, accessible, and reproducible content and an excellent customer experience.

*Nanoscale Advances* is the first journal by the Royal Society of Chemistry that is Gold Open Access from launch, and the first in a series of new launches that aim to support our transition towards open access publishing, in line with the rapid changes in the publishing landscape. In July 2021, *Nanoscale Advances* introduced article processing charges (APCs) for articles that are accepted following peer review.^3^ The competitive APC is in place to support a sustainable open access business model. We believe that open access publishing needs to be available to everyone, regardless of their economic background, thus discounts and waivers are available for authors, depending on their individual circumstances. The Royal Society of Chemistry is a not-for-profit publisher, so our APCs not only cover the high-quality peer review process for the journal and the maintenance of our publication platforms, but also support the services and resources the Royal Society of Chemistry provides to the community.^4^ These range from grants and awards, to tailored support for chemistry teachers and people working in industry.

Corresponding authors who pay an APC to publish in *Nanoscale Advances* are offered a year’s free affiliate membership of the Royal Society of Chemistry, so they can take advantage of some of the benefits we offer, such as membership of subject interest groups, career advice, and mentoring.

## Working with the nanoscale journal family

The nanoscale journal family at the Royal Society of Chemistry provides full coverage of interdisciplinary advances in nanoscience and nanotechnology. The family of journals are *Nanoscale Horizons*, *Nanoscale*, and *Nanoscale Advances*, and these are all part of a collaborative venture between the Royal Society of Chemistry and a leading nanoscience institute, the National Center for Nanoscience and Technology (NCNST) in Beijing, China.

Each journal covers the breadth of topics within nanoscience and nanotechnology, but each has its own distinguishing features so you can choose the journal that is right for you and your research.

*Nanoscale Horizons* aims to publish ground-breaking work representing a new concept or a new way of approaching a problem. *Nanoscale* and *Nanoscale Advances* aims to publish original, insightful, and significant papers, which are also reproducible, but the key criterion for publication in *Nanoscale* is novelty. The main difference between *Nanoscale* and *Nanoscale Advances* is the general significance and broad appeal of the work. Manuscripts of broad general interest are suitable for *Nanoscale* and those of a more specialised nature might be suited for *Nanoscale Advances*. Most importantly, *Nanoscale Advances* offers you Gold Open Access publication with a choice of CC-BY or CC-BY-NC licenses. This might be of particular importance to you due to personal preference or because of mandates from your funder.

We offer an efficient manuscript transfer service, where appropriate. Our hope is that no matter which journal you submit to, you will find the most appropriate home for your work with the minimum hassle. To facilitate this, *Nanoscale* and *Nanoscale Advances* have a shared Editorial Board, so the same high profile Associate Editors handle manuscripts for both journals. If you submit to *Nanoscale* and are offered a transfer to *Nanoscale Advances*, the same Associate Editor will handle your article in both journals. This reduces delays and ensures an efficient transfer of the history of your article, including any reviewer comments. In addition, several of our Associate Editors also act as Scientific Editors on *Nanoscale Horizons*, helping to provide a complete overview of the family to ensure we are providing you with a consistent experience.

## Looking ahead

We have big plans for the future and we invite you to be a part of them. A new themed collection programme will be launched throughout 2022 to feature aspects of nanoscience and nanotechnology where particularly exciting developments are taking place.

Professor Dirk Guldi, Editor-in-Chief: “*Nanoscience and nanotechnology have gained significant momentum in research activity. Considering the transdisciplinary nature of such research, it is important to have a versatile platform to disseminate new advances in the field. To this end, we not only turn to our committed authors and express a deep gratitude for their valuable contributions, but also give a warm welcome to our new readers/authors.*”

We are committed to developing and taking *Nanoscale Advances* forward so that the journal fully meets the needs of our authors and readers. We always welcome comments, suggestions, and feedback, so please do contact us at nanoscaleadvances-rsc@rsc.org with your views and feedback.

Professor Dirk Guldi, Editor-in-Chief

ORCID: http://orcid.org/0000-0002-3960-1765

Dr Anna Rulka, Executive Editor

ORCID: http://orcid.org/0000-0002-3236-9801

Dr Hannah Kerr, Deputy Editor

ORCID: http://orcid.org/0000-0002-2450-126X

Dr Alexander Whiteside, Development Editor

ORCID: http://orcid.org/0000-0002-2450-126X

## Supplementary Material
